# Cancer survivorship challenges of rural older adults: a qualitative study

**DOI:** 10.1186/s12885-023-11395-z

**Published:** 2023-09-28

**Authors:** Evelyn Arana-Chicas, Laura M. Hincapie Prisco, Saloni Sharma, Fiona Stauffer, Martha McGee, Serge Dauphin, Makiko Ban-Hoefen, Jaime Navarette, Jason Zittel, Ana Paula Cupertino, Allison Magnuson, Karen M. Mustian, Supriya G. Mohile

**Affiliations:** 1 James P. Wilmot Cancer Institute, University of Rochester Medical Center, Rochester, NY USA; 2 Division of Supportive Care in Cancer, Department of Surgery, University of Rochester Medical Center, Rochester, NY USA; 3 Geriatric Oncology Research Group, University of Rochester Medical Center, Rochester, NY USA; 4 Specialized Oncology Care & Research for our Elders Board Patient and Caregiver Advocate Board (SOCAREboard), University of Rochester Medical Center, Rochester, NY USA; 5 Arnot Medical Center, Rochester, NY USA; 6 Rochester General Hospital, Rochester, NY USA; 7 Department of Surgery, University of Rochester Medical Center, Rochester, NY USA; 8 Division of Hematology/Oncology, Department of Medicine, University of Rochester Medical Center, New York, USA

**Keywords:** Older adults, Rural populations, Cancer survivorship, Geriatric oncology

## Abstract

**Background:**

Although research has advanced the field of oncologic geriatrics with survivors to assess their cancer-related needs and devise patient-centered interventions, most of that research has excluded rural populations. This study aimed to understand the survivorship challenges and recommendations in the perspective of rural older adults.

**Methods:**

This was a qualitative study that explored the survivorship challenges and recommendations of rural older adults who have completed curative intent chemotherapy for a solid tumor malignancy in the 12 months prior to enrollment in the present study.

**Results:**

Twenty-seven older adult survivors from rural areas completed open-ended semi-structured interviews. The mean age was 73.4 (*SD* = 5.0). Most participants were non-Hispanic White (96.3%), female (59.3%), married (63.0%), and had up to a high school education (51.9%). Rural older survivors reported a general lack of awareness of survivorship care plans, communication challenges with healthcare team, transportation challenges, financial toxicity, psychological challenges, and diet and physical challenges. Rural older survivors recommend the provision of nutritional advice referral to exercise programs, and social support groups and for their healthcare providers to discuss their survivorship plan with them.

**Conclusions:**

Although study participants reported similar survivorship challenges as urban older adult survivors, additional challenges reported regarding transportation and consideration of farm animals have not been previously reported. Heightened awareness of the survivorship needs of rural older adults may result in better survivorship care for this population.

## Background

The number of older cancer survivors is increasing as treatment and diagnosis improves clinical outcomes [1–5]. Currently, 64% of cancer survivors are aged 65+; by 2040, almost 50% will be aged 75+, including 18% aged 85+ [6]. However, this gain in years of life is not followed with gains in quality of life (QOL). Older cancer survivors have a high prevalence of functional and cognitive impairment, comorbidities, and geriatric syndromes [7–10]. The National Academy of Medicine (NAM; previously Institute of Medicine) has stated that the needs of this population “are complex due to functional and cognitive impairment, multiple coexisting diseases, increased side effects from treatment, and greater need for social support.” [11, 12].

Older cancer survivors experience physical and cognitive impairments following curative-intent chemotherapy [13–17]. Symptomatic toxicities (e.g., fatigue, pain) further enhance physical and cognitive impairments, hospitalizations, and mortality [18–24]. Older patients report more interference with function from symptomatic toxicities than younger patients [25–30]. Older patients with cancer consistently prioritize recovery of their physical and cognitive function after chemotherapy as two areas of importance [31–33].

Rural-dwelling individuals in general tend to be older, of lower socioeconomic status, less likely to have transportation, and less educated compared to those in urban settings [34, 35]. Although research has advanced the field of oncologic geriatrics with survivors to assess their cancer-related needs and devise patient-centered interventions, most of that research has focused on urban populations [36–41]. A study analyzing the 2006–2010 National Health Interview Survey found that rural adult survivors reported worse health in all domains, were more likely to report fair/poor health, and had more comorbidities than urban cancer survivors [42]. While that analysis focused mostly on all age groups of rural cancer survivors, identifying the health and health care needs and opportunities to intervene with rural older adult survivors is essential. Including these populations in research will help us identify areas to intervene in their post-treatment survivorship care (e.g., care coordination, quality of life). Therefore, this study aimed to understand the survivorship challenges and recommendations in the perspective of rural older adults.

## Methods

### Study design and population

This was a qualitative study that explored the survivorship challenges and recommendations of rural older adults who have completed curative intent chemotherapy for a solid tumor malignancy in the 12 months prior to enrollment in the present study. Participants underwent a semi-structured interview (conducted by authors EAC and SS), which closely followed domains developed by Krok-Schoen et al. (Table 1) [43] that addresses perspectives of survivorship care among older cancer survivors.

**Table 1:** Domains of the interview guide that explored survivorship challenges of rural older adults

Interview Domain	Domain Description	Example of interview question(s)
1. Development of a survivorship care plan	Participants were asked whether they developed with their care team, or were provided with, a cancer survivorship care plan (SCP). SCP was defined to the participant as “A summary of your treatment, together with recommendations for follow-up care and leading a healthy lifestyle.”	Tell me whether you developed, or were provided with, a survivorship care plan with your oncologist or physician after completing cancer treatment.
2. Communication of survivorship care	Participants shared their experiences with communication with their healthcare providers after completing curative-intent chemotherapy.	What conversations did you have with your healthcare team about what you could expect as someone who has completed chemotherapy? What are your impressions of how communication is/was going between your oncology team and primary care doctor?
3. Survivorship care challenges	Participants shared any challenges they may have experienced during their post-treatment phase (e.g., side effects, transportation, etc.)	Did you experience any challenges during post-treatment cancer care? If yes, tell me about them.
4. Supportive Network	Participants shared whether they have a supportive network/individual, and if they do, their role during cancer treatment and after the conclusion of cancer treatment.	Please tell me about your social support network. What has been the role of family and/or friends during cancer treatment? How about after your cancer treatment ended?
5. Recommendations to survivorship care	Participants shared recommendations to improving their post-treatment survivorship care.	Being a person who has completed chemotherapy, do you have any advice for how to improve your survivorship care experience?

Purposive sampling was used to recruit participants who met the following eligibility criteria: (1) Rural-dwelling living in a zip code designated area per the HRSA Federal Office of Rural Health Policy (FORHP) or the Centers for Medicare and Medicaid Services (CMS) [44], (2) age 65 or older, (3) have a diagnosis of a solid tumor malignancy, (4) completed curative-intent chemotherapy in the past 12 months, and (5) has no future chemotherapy planned. Participants were recruited from the Wilmot Cancer Institute (WCI) and affiliate clinics and Arnot Medical Center using these methods: (1) clinic staff screened for eligible patients from the oncology clinic schedules (with site oncologist permission) who have completed curative-intent chemotherapy for cancer in the past 12 months and presented the study to the patient; (2) clinic staff identified prospective subjects through electronic medical records and clinic staff would call potential subjects to present the study; and (3) research staff sent MyChart messages to potentially eligible participants with information about the study. This study was approved by the University of Rochester Medical Center Research Subjects Review Board and Arnot Medical Center Institutional Review Board.

### Study procedure and data collection

After reviewing the study aims and risks and benefits to participation, informed verbal consent was obtained from all participants. Consent was obtained either in person during a participant’s clinic visit or via telephone. Demographic information collected included age, gender, race, marital status, education, and annual income. Tumor and Treatment characteristics were also collected that had cancer diagnosis type, cancer diagnosis date, and tumor stage. Semi-structured interviews were conducted via telephone privately with the interviewer and participant. After completing the interview, each participant was compensated with a $50 pre-paid Visa card. EAC and SS completed interviews which lasted ~ 40 min and were audiotaped.

### Analysis

Descriptive statistics of study participant characteristics and demographics were assessed. Interview transcripts were professionally transcribed in their entirety and imported into the MAXQDA software analytic program for analysis. EAC, LHP, and FS conducted an inductive thematic analysis to code and analyze the data collected. An iterative process was used to create a codebook that included details about code definitions. EA and LHP reviewed the codes together to organize them into overarching themes.

## Results

Twenty-seven older adult survivors from rural areas completed open-ended semi-structured interviews. The mean age was 73.4 (*SD* = 5.0; Table 2). Most participants were non-Hispanic White (96.3%), female (59.3%), married (63.0%), and had up to a high school education (51.9%). All participants had health insurance and 51.9% had an annual income <$50k. Most participants were diagnosed with stage III (69.6%) cancer, and the majority were diagnosed with gastrointestinal cancers (56.6%). All participants had at least one comorbidity, the most common of which were high blood pressure and arthritis (both at 70.0%).

**Table 2:** Patient characteristics (n = 27)

	Mean (SD) or N (%)
Age (years)	73.4 (5.0)
Gender	
Female	16 (59.3%)
Race or ethnicity	
Non-Hispanic White Hispanic	26 (96.3%) 1 (3.7%)
Marital Status	
Single or never married Married or domestic partnership Separated, widowed, or divorced	1 (3.7%) 17 (63.0%) 9 (33.3%)
Education	
Less than high school High school graduate Some college or above	2 (7.4%) 14 (51.9%) 11 (40.7%)
Income	
≤US $50,000 >US $50,000 Declined to answer	14 (51.9%) 12 (44.4%) 1 (3.7%)
Cancer type	
Breast Gastrointestinal Genitourinary Gynecologic Lung	2 (8.7%) 13 (56.6%) 2 (8.7%) 1 (4.3%) 5 (21.7%)
Cancer stage	
I II III	2 (8.7%) 5 (21.7%) 16 (69.6%)
Comorbidities	
Arthritis Circulation Trouble in Arms or Legs Depression Diabetes Emphysema High Blood Pressure Heart Disease Osteoporosis Stomach/Intestinal Disorders	17 (70.0%) 5 (20.8%) 7 (29.2%) 5 (20.8%) 5 (20.8%) 17 (70.0%) 4 (16.7%) 8 (33.3%) 8 (33.3%)

The following themes were identified: (1) Provision of a survivorship care plan, (2) Communication of survivorship care, (3) Diet and physical challenges, (4) Psychological challenges; 6) Transportation Challenges. Recommendations included: (1) Referral to a dietitian/nutritionist and (2) More information on exercise. Below is a summary of the themes with brief descriptions and representative quotes (also summarized in Table 3).

### Provision of a survivorship care plan (SCP)

When asked whether they developed a survivorship care plan with their healthcare team, most participants did not recall receiving an SCP.

Participant 7 (82-year-old male): *Neither doctor gave me any particular path to follow after the treatment.*

Participant 15 (80-year-old female): *No, not really. He [oncologist] said to me you’re pretty independent. You seem to be relatively healthy*.

Participant 13 (79-year-old female): P: *No, I don’t think so. He [oncologist] does keep me up on my appointments, though.*

Several participants recalled receiving paperwork but reported being overwhelmed and having medical terms they did not understand. Some participants also reported that they needed someone to sit with them to discuss the paperwork that they received.

Participant 10 (83-year-old female): *All the paperwork and everything that you get mentioned maintaining healthy lifestyle, healthy diet, but as far as actual discussion, no.*

Participant 25 (65-year-old male): *They overwhelm you with all this stuff [paperwork] and you start reading – a lot of it has such terms that you don’t understand.*

Participant 29 (78-year-old male): *When you look at the paperwork that you get when you leave the visits, they’re generally very complete and they make recommendations, but no one sat down with me and said, ‘Here are the things that you may confront, here’s how we would recommend you handle it.‘*

### Communication regarding survivorship care

All participants shared their communication experiences with their providers. Participants reported that their healthcare providers were not taking the time to consider their daily experiences managing farm animals. Provider-level barriers included the lack of continuity of care between the cancer care team with PCP and vice-versa.

Participant 1 (76-year-old male): *He [the surgeon] told me he wanted to operate on the next Monday, and I told him I didn’t think I could get rid of the cows that fast because I’m a dairy farmer and I had 60 cows to milk every day. He said get somebody to take care of the cows. He didn’t care what I did and didn’t give me time to think about anything.*

Participant 29 (78-year-old male): *Our life is very centered around the animals. I don’t think that they [providers] know that.*

Most participants assumed that their primary care provider (PCP) is communicating with their cancer care team electronically, although some of them have not talked to their PCP in a while. One patient reported acting as a messenger between them to ensure his PCP received information regarding his health.

Participant 33 (72-year-old female): *I’m assuming she [PCP] can access my medical information, but I really haven’t talked to her about it [cancer].*

Participant 15 (69-year-old female): *Everything is online, so I would assume he [PCP] gets all the information that everyone else does.*

Participant 9 (72-year-old male): *My primary care doctor wanted more information from him [oncologist] because he felt like he wasn’t getting enough information. He [PCP] had me give them messages to make sure he was getting the information.*

### Diet and physical challenges

Participants describe not having an appetite and food not tasting the same, even after completing their cancer treatment. One 82-year old female participant said: *I don’t have the appetite I used to have. My weight has gone down quite a bit and things still don’t taste right to me*. Participants also described losing their physical strength and feeling fatigued from their treatment.

Participant 1 (76-year-old male): *I’d like to get back to work, but I just can’t do a lot. I was born on this farm and I kind of kept my body young. I’ve always worked, I’ve always been active. I’d like to get things done the way they were, but then when you’re 76 years old I guess that don’t happen.*

Participant 9 (72-year-old male): *The chemotherapy actually drained me so bad I have no ambition to do anything and I felt like I couldn’t do anything; I was exhausted and it was an effort for me to even walk.*

### Transportation

Many patients reported being unable to drive and needing to rely on someone to take them to their cancer care appointments. Some also shared that they were not comfortable driving far out of their hometown. Some participants did report that although they live far from Rochester, they were able to handle the drive, with some even coordinating overnight stay with family members who live closer to the city.

Participant 1 (76-year-old male): *I’ve been here in this town so long and I don’t even know where anything is. I’m not a good driver. I don’t want to get in lot of traffic and stuff.*

Participant 3 (76-year-old female): *He [my son] took me to all those appointments and I have a friend that [also] takes me, because I won’t drive to the city.*

Participant 33 (71-year-old female): *What we would do because our daughter lives out there [in Rochester], we would come out the night before, spend the night, have the treatment, and then drive home after treatment.*

### Psychological challenges

A number of participants reported regret with undergoing chemotherapy, citing side effects were more intense than they thought it would be. Some patients also shared their concern that their cancer might return.

Participant 9 (72-year-old male): *There were all sorts of issues with the chemotherapy that I feel*.

*like if I have to go through it again, I don’t know that I would want to. I think I would prefer the*.

*alternative. That’s how bad it is.*

Participant 20 (72-year-old female): *That first week after treatment, I felt like if this is what it is going to be like I think I made a mistake.*

Participant 10 (82-year-old female): *I wonder what’s ahead, seeing as I know this is a cancer*.

*that is apt to return, that’s at the back of my mind.*

### Financial toxicity

Some participants reported financial challenges with paying medical bills related to their cancer treatments and medications. One patient, a dairy farmer, had to sell his cows and now has no income and is struggling financially.

Participant 1 (76-year-old male): *I’ve been on this farm 73 years. I sold my cows. There is no income coming in, and I got 400 acres here and I got bills to pay.*

Participant 9 (72-year-old male): *I take Creon and I’ll have to take it for the rest of my life after the Whipple procedure. It costs me around $425 a month.*

Participant 19 (71-year-old female): *My one bill from chemo was over $3,000. They knocked it down to like $900 and I’m paying them like a hundred dollars a month.*

### Recommendations to improve survivorship health

Several participants reported wanting access to a dietician and nutritional advice. One participant mentioned his doctor told him to eat anything he wanted.

Participant 14 (70-year-old female): *I think it would have been good for me to have access to a good dietitian, who would have been able to work with me with my food allergy issues.*

Participant 1 (76-year-old male): *I asked about diet but the doctors here don’t seem to worry too much about that. He [doctor] told me to eat whatever I want.*

Several participants also reported wanting to exercise more and wanted access to specific exercise plans. One participant reported thinking that her doctors did not provide her with an exercise plan because she is already a physically active person.

Participant 4 (65-year-old male): *I’ve actually been thinking of asking is there any exercises that maybe I can start. You know, taking care of my body exercise-wise.*

Participant 17 (82-year-old female): *I’ve been a very active person and I know quite a bit about nutrition, so I guess in my case, maybe they [healthcare team] thought it wasn’t needed.*

Participants also would like referrals to cancer survivorship groups, citing loneliness and a need to talk to someone.

Participant 19 (71-year-old female): *Possibly a support group. We had talked about this one time actually at the oncologist’s office, but it never happened.*

Participant 10 (82-year-old female): *Just that I have somebody to talk to*. (82, female)

Participants also suggesting having someone from their healthcare team talk them through their survivorship plan.

Participant 9 (72-year-old male): *The oncology center gave me all these handouts but I was gonna recommend that someone go through it with you in person with caregiver present.*

Participant 20 (72-year-old female): *We didn’t sit down and hammer it [survivorship plan] out. I’ve gotten so much paperwork since this whole thing started that’s hard to even keep track of what I’ve got and what I don’t have. There is no discussion set up.*

**Table 3:** Themes and representative quotes that emerged from thematic analysis (n = 27)

Themes	Example Quotations
**Provision of a survivorship care plan**	
No recall of receiving a SCP	Participant 7 (82-year-old male): *Neither doctor gave me any particular path to follow after the treatment.* Participant 15 (80-year-old female): *No, not really. He [oncologist] said to me you’re pretty independent. You seem to be relatively healthy*. Participant 13 (79-year-old female): P: *No, I don’t think so. He [oncologist] does keep me up on my appointments, though.* Participant 9 (72-year-old male): *The oncology center gave me all these handouts, but I was going to recommend that someone go through it with you in person with caregiver present and before you have the chemo as well.*
Overwhelmed with too much paperwork	Participant 10 (83-year-old female): *All the paperwork and everything that you get mentioned maintaining healthy lifestyle, healthy diet, but as far as actual discussion, no.* Participant 25 (65-year-old male): *They overwhelm you with all this stuff [paperwork] and you start reading – a lot of it has such terms that you don’t understand.* Participant 29 (78-year-old male): *When you look at the paperwork that you get when you leave the visits, they’re generally very complete and they make recommendations, but no one sat down with me and said, ‘Here are the things that you may confront, here’s how we would recommend you handle it.‘*
No discussions on long-term effects of treatment	Participant 10 (82-year-old female): *We didn’t really discuss it [expectations after treatment]; it’s like ‘You’re good’.* Participant 15 (69-year-old female): *Well, we did not really discuss what’s down the road because we really can’t tell. Everybody’s different.* Participant 1 (76-year-old male): *I guess I assumed when I was done with chemo, after a period of time the side effects were going to go away. Well, they’re not going away.*
**Communication regarding survivorship care**	
Lack of consideration for personal farm animals	Participant 1 (76-year-old male): *He [the surgeon] told me he wanted to operate on the next Monday, and I told him I didn’t think I could get rid of the cows that fast because I’m a dairy farmer and I had 60 cows to milk every day. He said get somebody to take care of the cows. He didn’t care what I did and didn’t give me time to think about anything.* Participant 29 (78-year-old male): *Our life is very centered around the animals. I don’t think that they [providers] know that.*
Lack of communication between PCP and cancer care team	Participant 33 (72-year-old female): *I’m assuming she [PCP] can access my medical information, but I really haven’t talked to her about it [cancer].* Participant 15 (69-year-old female): *Everything is online, so I would assume he [PCP] gets all the information that everyone else does.* Participant 9 (72-year-old male): *My primary care doctor wanted more information from him [oncologist] because he felt like he wasn’t getting enough information. He [PCP] had me give them messages to make sure he was getting the information.*
Learning of cancer diagnosis through MyChart	Participant 17 (82-year-old female): *MyChart is wonderful in one way in that you can see things right up front and you can communicate with your doctors and they get back to you right away. But on the other hand, it’s a little scary when you see, oh, I have cancer, you know?* Participant 14 (70-year-old female): *The other thing that I just thought was horrible was I’ve been waiting for the results of the biopsy and they said, you know, within 48 h. So, it was past 48 h, and I hadn’t gotten anything, but 15 min after their office closed, I got those results in MyChart. And then I’m looking at them and I’m thinking I know what it says, but then I’m like wait a minute, you know, I’m not medically trained for anything here.*
**Diet and Physical Challenges**	
Lack of appetite/food tasting different	Participant 21 (82-year-old female): *I don’t have the appetite I used to have. My weight has gone down quite a bit and things still don’t taste right to me*. Participant 19 (71-year-old female): *After the chemotherapy, it was just what I could eat and they [healthcare team] were encouraging me to, but things didn’t taste good.*
Difficulty with physical activity	Participant 10 (82-year-old female): *With the oxygen, if I have to go to my pulmonary doctor, which is the 4th floor and it’s quite a walk, I had somebody go with me to carry the oxygen.* Participant 1 (76-year-old male): *I’d like to get back to work, but I just can’t do a lot. I was born on this farm and I kind of kept my body young. I’ve always worked, I’ve always been active. I’d like to get things done the way they were, but then when you’re 76 years old I guess that don’t happen.*
**Transportation Challenges**	
Reliance on someone to take them to appointments	Participant 1 (76-year-old male): *I’ve been here in this town so long and I don’t even know where anything is. I’m not a good driver. I don’t want to get in lot of traffic and stuff.* Participant 3 (76-year-old female): *He [my son] took me to all those appointments and I have a friend that [also] takes me, because I won’t drive to the city.* Participant 23 (73-year-old female): *I’m not comfortable driving because I’ve lost some of my peripheral vision. I have to have a ride wherever I go.*
Coordinate overnight stay with family members in the city	Participant 33 (71-year-old female): *What we would do because our daughter lives out there [in Rochester], we would come out the night before, spend the night, have the treatment, and then drive home after treatment.*
**Psychological Challenges**	
Medical regret	Participant 9 (72-year-old male): *There were all sorts of issues with the chemotherapy that I feel like if I have to go through it again, I don’t know that I would want to. I think I would prefer the alternative. That’s how bad it is.* Participant 20 (72-year-old female): *That first week after treatment, I felt like if this is what it is going to be like I think I made a mistake.*
Fear of cancer recurrence	Participant 10 (82-year-old female): *I wonder what’s ahead, seeing as I know this is a cancer that is apt to return, that’s at the back of my mind.* Participant 4 (66-year-old male): *Every time a scan comes up, for a couple of weeks I get a little nervous.* Participant 25 (65-year-old male): *There was ground glass opacity in one of my lungs in the upper lobe, she [the doctor] thought that it could be either metastatic lung cancer and that destroyed me.*
**Financial Toxicity**	
Financial Toxicity Present	Participant 1 (76-year-old male): *I’ve been on this farm 73 years. I sold my cows. There is no income coming in, and I got 400 acres here and I got bills to pay.* Participant 9 (72-year-old male): *I take Creon and I’ll have to take it for the rest of my life after the Whipple procedure. It costs me around $425 a month.* Participant 19 (71-year-old female): *My one bill from chemo was over $3,000. They knocked it down to like $900 and I’m paying them like a hundred dollars a month.*
**Recommendations to improve survivorship care**	
Referral to a dietitian	Participant 14 (70-year-old female): *I think it would have been good for me to have access to a good dietitian, who would have been able to work with me with my food allergy issues.* Participant 1 (76-year-old male): *I asked about diet but the doctors here don’t seem to worry too much about that. He [doctor] told me to eat whatever I want.* Participant 9 (72-year-old male): *Maybe nutrition info I would have liked. I know I should be eating nutritious meals.*
Information on exercise	Participant 4 (65-year-old male): *I’ve actually been thinking of asking is there any exercises that maybe I can start. You know, taking care of my body exercise-wise.* Participant 10 (82-year-old female): *I’d like to do some more walking. I think it would help me.* Participant 17 (82-year-old female): *I’ve been a very active person and I know quite a bit about nutrition, so I guess in my case, maybe they [healthcare team] thought it wasn’t needed.*
Referral to Cancer Support Groups	Participant 19 (71-year-old female): *Possibly [referral to] a support group. We had talked about this one time actually at the oncologist’s office, but it never happened.* Participant 10 (82-year-old female): *I just want somebody to talk to.* (82, female)
Discuss survivorship plan with patient	Participant 9 (72-year-old male): *The oncology center gave me all these handouts but I was gonna recommend that someone go through it with you in person with caregiver present.* Participant 20 (72-year-old female): *We didn’t sit down and hammer it [survivorship plan] out. I’ve gotten so much paperwork since this whole thing started that’s hard to even keep track of what I’ve got and what I don’t have. There is no discussion set up.*

## Discussion

This qualitative study sought to understand the cancer survivorship needs of rural older adults aged 65 + who have completed curative-intent chemotherapy in the past 12 months. Results indicated a variety of challenges encountered, including a general lack of awareness of survivorship care plans, communication challenges with the healthcare team, transportation challenges, psychological challenges, and diet and physical challenges.

Most participants did not recall receiving a survivorship care plan (SCP) from their healthcare team, and the few that did receive a SCP reported being overwhelmed with all the paperwork and health care providers not taking the time to talk them through the SCP. This is corroborated by previous research reporting that few older adults develop a SCP and those that do report poor communication with their healthcare providers [43, 45]. Past research also suggests that survivors need assistance in implementing the recommendations outlined in their SCP (e.g., to schedule and attend follow-up appointments, behavior change via diet and/or exercise, etc.) [46, 47]. Although there is controversy about SCPs [48], patients should be educated on recommendations for follow-up care and leading a healthy lifestyle.

In addition, participants reported providers not taking the time to consider the impact that their patients’ rural lifestyle may have on their cancer care. Participants reported caring for personal farm animals but reported providers not considering their animals’ care. This in turn, can lead to a reduced QOL if the animals have to be sold and possibly to financial toxicity if the farm animals were used as a source of income. Moreover, there appears to be a lack of communication between the patient’s cancer care team and the PCP. Previous research has also shown that PCPs are often disconnected from the cancer care team due to ineffective communication and poor integration of treatment plans [49, 50]. In addition, as comorbidities may negatively impact cancer survival outcomes [51], PCPs are necessary throughout cancer survivorship. Given that older adult [] and rural individuals [52,53] are more likely to have comorbidities than younger adults and urban individuals, PCPs need a more active role in managing comorbidities and side effects of older cancer survivors.

Although all participants could attend their cancer care appointments, they had to rely on someone to drive them to their appointments. Participants did not mention having trouble finding someone to take them to their appointments and may be associated with them having a strong social support network. Research has shown that rural cancer survivors lack reliable and affordable transportation which may prevent them from accessing timely and effective cancer care [54, 55]. Future research should look at transportation challenges from more historically marginalized rural older adult populations (e.g., racial/ethnic minorities, lower income, etc.).

Moreover, medical regret undergoing chemotherapy and fear of cancer recurrence was reported by some participants in this study. Although research has shown low levels of decision regret for older female breast cancer survivors [56], future research should explore the extent of decisional regret in a larger sample of rural older adult survivors.

Not surprisingly, participants recommended providing nutritional advice and referral to exercise programs. Nutrition is important for older adult survivors for numerous reasons, including needing help managing issues with their gastrointestinal tract as a result of treatment, interest in behavioral diet strategies to reduce the risk of cancer recurrence, and to help manage comorbidities [57]. Moreover, research suggest that physical activity interventions are safe and effective in older cancer survivors [58]. Older patients with cancer consistently prioritize recovery of their physical function after chemotherapy [59, 60].

Most of our results are similar to that reported by low-income urban cancer survivors. However, transportation barriers, while experienced by both rural and urban individuals, differ for both. Most participants in our study had a car but were not able to drive the long distance to their healthcare appointments. Urban low-income individuals experience different transportation barriers such as not having any form of transportation or monetary resources to take the bus go to their healthcare visit. The lack of consideration of farm animals was also unique to rural populations [62–64]. The common barriers between both urban and rural might be more related to socioeconomic status than rurality – a quantitative study with a larger sample size that investigates this would be worthwhile.

This study has several limitations. The sample was racially homogeneous, with most of our sample self-identifying as non-Hispanic White, which limits generalizability to other populations. Given that most agricultural farmworkers (78%) are Hispanic, [64] more work needs to be done to recruit this population and other racially and ethnically diverse rural populations to learn about their unique survivorship needs. Local partnerships with farmworker coalitions and with local rural clinics and organizations can assist in the identification and recruitment of these underserved populations. Moreover, most of our sample had a strong social support network. It is possible that rural older adult survivors who lack a social support network and/or are a racial and/or ethnic individuals, would have additional survivorship challenges than are reported in this study. Future research should consider understanding the cancer survivorship challenges of these populations.

## Conclusions

This qualitative study sought to understand the cancer survivorship needs of rural older adults.

Results indicated various challenges, including a general lack of awareness of survivorship care plans, communication challenges with healthcare team, transportation challenges, psychological challenges, and diet and physical challenges. Although our participants reported similar survivorship challenges as urban older adult survivors, additional challenges reported regarding transportation and consideration of farm animals have not been reported in the literature. Participants recommend the provision of nutritional advice, referral to exercise programs, and social support groups and for their healthcare providers to discuss their survivorship plan with them. Healthcare providers should consider the unique needs of rural older adults and educate them on recommendations for follow-up survivorship care and leading a healthy lifestyle. Future research should explore the challenges of rural older survivors with more marginalized identities (e.g., racial/ethnic minority, low income, etc.).

## Data Availability

The datasets used and/or analyzed during the current study are available from the corresponding author on reasonable request.

## References

[1] Rowland JH, Bellizzi KM (2014). Cancer survivorship issues: life after treatment and implications for an aging population. J Clin Oncol.

[2] Parry C, Kent EE, Mariotto AB, Alfano CM, Rowland JH (2011). Cancer survivors: a booming population. Cancer Epidemiol Biomarkers Prev.

[3] de Moor JS, Mariotto AB, Parry C, Alfano CM, Padgett L, Kent EE (2013). Cancer survivors in the United States: prevalence across the survivorship trajectory and implications for care. Cancer Epidemiol Biomarkers Prev.

[4] Shapiro CL (2018). Cancer Survivorship. N Engl J Med.

[5] Miller KD, Nogueira L, Mariotto AB, Rowland JH, Yabroff KR, Alfano CM (2019). Cancer treatment and survivorship statistics, 2019. CA Cancer J Clin.

[6] Bluethmann SM, Mariotto AB, Rowland JH (2016). Anticipating the silver tsunami: prevalence trajectories and Comorbidity Burden among Older Cancer Survivors in the United States. Cancer Epidemiol Biomarkers Prev.

[7] Mohile SG, Xian Y, Dale W, Fisher SG, Rodin M, Morrow GR, Neugut A, Hall W (2009). Association of a cancer diagnosis with vulnerability and frailty in older Medicare beneficiaries. J Natl Cancer Inst.

[8] Mohile SG, Fan L, Reeve E, Jean-Pierre P, Mustian K, Peppone L (2011). Association of cancer with geriatric syndromes in older Medicare beneficiaries. J Clin Oncol.

[9] Williams GR, Mackenzie A, Magnuson A, Olin R, Chapman A, Mohile S (2016). Comorbidity in older adults with cancer. J Geriatr Oncol.

[10] Brauer ER, Ganz PA (2019). Health burden in cancer survivors: below the tip of the iceberg. Nat Reviews Clin Oncol.

[11] Nekhlyudov L, Levit L, Hurria A, Ganz PA (2014). Patient-centered, evidence-based, and cost-conscious cancer care across the continuum: translating the Institute of Medicine report into clinical practice. CA Cancer J Clin.

[12] Hurria A, Naylor M, Cohen HJ (2013). Improving the quality of cancer care in an aging population: recommendations from an IOM report. JAMA.

[13] Presley CJ, Han L, Leo-Summers L, Hurria A, Gross CP, Davidoff AJ (2019). Functional trajectories before and after a new cancer diagnosis among community-dwelling older adults. J Geriatr Oncol.

[14] Mandelblatt JS, Clapp JD, Luta G, Faul LA, Tallarico MD, McLendon TD (2016). Long-term trajectories of self-reported cognitive function in a cohort of older survivors of breast cancer: CALGB 369901 (Alliance). Cancer.

[15] Henderson TO, Ness KK, Cohen HJ (2014). Accelerated aging among cancer survivors: from pediatrics to geriatrics. Am Soc Clin Oncol Educational book Am Soc Clin Oncol Annual Meeting.

[16] Pergolotti M, Deal AM, Williams GR, Bryant AL, Bensen JT, Muss HB (2017). Activities, function, and health-related quality of life (HRQOL) of older adults with cancer. J Geriatr Oncol.

[17] Magnuson A, Lei L, Gilmore N, Kleckner AL, Lin FV, Ferguson R (2019). Longitudinal relationship between Frailty and Cognition in Patients 50 years and older with breast Cancer. J Am Geriatr Soc.

[18] Mohile SG, Heckler C, Fan L, Mustian K, Jean-Pierre P, Usuki K (2011). Age-related differences in symptoms and their interference with quality of life in 903 Cancer patients undergoing Radiation Therapy. J Geriatric Oncol.

[19] Sprod LK, Mohile SG, Demark-Wahnefried W, Janelsins M, Peppone L, Morrow GR (2012). Exercise and Cancer treatment symptoms in 408 newly diagnosed older Cancer Patients. J Geriatr Oncol.

[20] Given CW, Given B, Azzouz F, Kozachik S, Stommel M (2001). Predictors of pain and fatigue in the year following diagnosis among elderly cancer patients. J Pain Symptom Manag.

[21] Quinten C, Kenis C, Hamaker M, Coolbrandt A, Brouwers B, Lago LD (2018). The effect of adjuvant chemotherapy on symptom burden and quality of life over time; a preliminary prospective observational study using individual data of patients aged >/=70 with early stage invasive breast cancer. J Geriatr Oncol.

[22] Hurria A, Mohile S, Gajra A, Klepin H, Muss H, Chapman A (2016). Validation of a Prediction Tool for Chemotherapy Toxicity in older adults with Cancer. J Clin Oncol.

[23] Du XL, Osborne C, Goodwin JS (2002). Population-based assessment of hospitalizations for toxicity from chemotherapy in older women with breast cancer. J Clin Oncol.

[24] Nurgalieva Z, Liu CC, Du XL (2009). Risk of hospitalizations associated with adverse effects of chemotherapy in a large community-based cohort of elderly women with ovarian cancer. Int J Gynecol Cancer.

[25] Pandya C, Magnuson A, Dale W, Lowenstein L, Fung C, Mohile SG (2016). Association of falls with healthrelated quality of life (HRQOL) in older cancer survivors: a population-based study. J Geriatr Oncol.

[26] Fodeh SJ, Lazenby M, Bai M, Ercolano E, Murphy T, McCorkle R (2013). Functional impairments as symptoms in the symptom cluster analysis of patients newly diagnosed with advanced cancer. J Pain Symptom Manag.

[27] Jensen RE, Potosky AL, Moinpour CM, Lobo T, Cella D, Hahn EA (2017). United States Population-Based estimates of PatientReported Outcomes Measurement Information System Symptom and Functional Status reference values for individuals with Cancer. J Clin Oncol.

[28] Yates P, Miaskowski C, Cataldo JK, Paul SM, Cooper BA, Alexander K (2015). Differences in composition of Symptom clusters between older and younger oncology patients. J Pain Symptom Manag.

[29] Luciani A, Jacobsen PB, Extermann M, Foa P, Marussi D, Overcash JA (2008). Fatigue and functional dependence in older cancer patients. Am J Clin Oncol.

[30] Loh KP, Pandya C, Zittel J, Kadambi S, Flannery M, Reizine N (2017). Associations of sleep disturbance with physical function and cognition in older adults with cancer. Support Care Cancer.

[31] Fried TR, Bradley EH, Towle VR, Allore H (2002). Understanding the treatment preferences of seriously ill patients. N Engl J Med.

[32] Karuturi M, Wong ML, Hsu T, Kimmick GG, Lichtman SM, Holmes HM (2016). Understanding cognition in older patients with cancer. J Geriatr Oncol.

[33] Magnuson A, Wallace J, Canin B, Chow S, Dale W, Mohile SG (2016). Shared goal setting in Team-Based geriatric oncology. J Oncol Pract.

[34] Bollman RD, Reimer W, Demographics (2009). Employment, income, and networks: Differential characteristics of rural populations. J Agromed.

[35] Zahnd WE, Scaife SL, Francis ML (2009). Health literacy skills in rural and urban populations. Am J Health Beh.

[36] Loh KP, Abdallah M, Kadambi S, Wells M, Kumar AJ, Mendler JH (2021). Treatment decision-making in acute myeloid leukemia: a qualitative study of older adults and community oncologists. Leuk Lymphoma.

[37] Loh KP, Soto-Perez-de-Celis E, Duberstein PR, Culakova E, Epstein RM, Xu H (2021). Patient and caregiver agreement on prognosis estimates for older adults with advanced cancer. Cancer.

[38] Sedrak MS, Freedman RA, Cohen HJ, Muss HB, Jatoi A, Klepin HD et al. Older adult participation in cancer clinical trials: a systematic review of barriers and interventions. CA: a cancer journal for clinicians. 2021; 10.3322/caac.21638. 10.3322/caac.21638 PMC7854940 33002206

[39] Kadambi S, Loh KP, Dunne R, Magnuson A, Maggiore R, Zittel J (2020). Older adults with cancer and their caregivers - current landscape and future directions for clinical care. Nature reviews. Clin Oncol.

[40] Williams GR, Weaver KE, Lesser GJ, Dressler E, Winkfield KM, Neuman HB (2020). Capacity to provide geriatric Specialty Care for older adults in Community Oncology Practices. ” the Oncologist.

[41] Verduzco-Aguirre HC, Babu D, Mohile SG, Bautista J, Xu H, Culakova E (2021). Associations of uncertainty with psychological health and quality of life in older adults with advanced cancer. J Pain Symptom Manag.

[42] Weaver KE, Geiger AM, Lu L, Case D (2014). Rural-urban disparities in Health Status among US Cancer Survivors. Cancer.

[43] Krok-Schoen JL, Naughton MJ, Noonan AM, Pisegna J, DeSalvo J, Lustberg MB (2020). Perspectives of Survivorship Care Plans among older breast Cancer survivors: a pilot study. Cancer Control.

[44] U.S. Department of Health and Human Services. Defining Rural Population. Health Resources & Services Administration. 2018. Available at https://www.hrsa.gov/rural-health/aboutus/definition/index.html. Accessed June 10, 2023.

[45] Krok-Schoen JL, Fernandez K, Unzeitig GW, Rubio G, PAskett ED, Post DM (2019). Hispanic breast cancer patients’ symptom experience and patient-physician communication during chemotherapy. Supportive Care.

[46] Birken SA, Clary AS, Bernstein S, Bolton J, Tardif-Douglin M, Mayer DK (2018). Strategies for successful Survivorship Care Plan implementation: results from a qualitative study. J Oncol Pract.

[47] Stricker CT, Jacobs LA, Risendal B, Jones A, Panzer S, Ganz PA (2011). Survivorship care planning after the Institute of Medicine recommendations: how are we faring?. J Caner Surviv.

[48] Ganz P, Grunfeld E. Survivorship care plans: 2013 Quality Care Symposium.

[49] Sussman J, Baldwin L (2010). The interface of primary and oncology specialty care: from diagnosis through primary treatment. J Natl Cancer Inst Monographs.

[50] Sada Y, Street RL, Singh H, Shada R, Naik AD (2011). Primary care and communication in shared cancer care: a qualitative study. Am J Manag Care.

[51] Fowler H, Belot A, Ellis L, Maringe C, Luque-Fernandez MA, Njagi N, Navani N (2020). Comorbidity prevalence among cancer patients: a population-based cohort study of four cancers. BMC Cent.

[52] O’Connor A, Wellenius G (2012). Rural-urban disparities in the prevalence of diabetes and coronary heart disease. Public Health.

[53] Kumar V, Acanfora M (2001). Health status of the rural elderly. J Rural Health.

[54] Jiang C, Yabroff KR, Deng L, Wang Q, Perimebti S, Shapiro CL (2023). Transportation barriers, emergency room use, and mortality risk among US adults by cancer history. J Nat Cancer Inst.

[55] Wecholuk A, Parikh AA, Snyder RA (2022). The Road Less traveled: transportation barriers to Cancer Care Delivery in the Rural Patient Population. JCO Oncol Pract.

[56] Karuturi M, Lei X, Shen Y, Giordano SH, Swanick CW, Smith BD (2019). Long-term decision regret surrounding systemic therapy in older breast cancer survivors: a population-based survey study. J Geriatr Oncol.

[57] Kleckner A, Magnuson A (2022). The nutritional needs of older cancer survivors. J Geriatr Oncol.

[58] Klepin H, Mohile S, Mihalko S (2013). Exercise for older cancer patients: feasible and helpful?. Interdiscip Top Gerontol.

[59] Duan-Porter W, Cohen HJ, Demark-Wahnefried W, Sloane R, Pendergast JF, Snyder DC (2016). Physical resilience of older Cancer Survivors: an emerging Concept. J Geriatr Oncol.

[60] Pandya C, Magnuson A, Flannery M, Zittel J, Duberstein P, Loh KP (2019). Association between symptom burden and physical function in older patients with cancer. J Am Geriatr Soc.

[61] Pisegna JL, BrintzenhofeSzoc K, Shahrokni A, Canin B, Plotkin E, Boehmer LM (2023). Differences in urban and suburban/rural settings regarding care provision and barriers of cancer care for older adults during COVID–19. Support Care Cancer.

[62] Dulko D, Pace CM, Dittus KL, Sprague BL, Pollack LA et al. Barriers and facilitators to implementing cancer survivorship care plans. 2013. 40(6):575–80. 10.1188/13.ONF.575-580 PMC4501016 24161636

[63] Argenbright K, Berry E (2021). Innovative Cancer Survivorship Services for Rural and Underserved Communities. JNCI.

[64] National Center for Farmworker Health, Inc. Facts About Agricultural Workers. Jan 2022. Available at https://www.ncfh.org/facts-about-agricultural-workers-fact-sheet.html#:~:text=Sixty%2Dsix%20%%20of%20crop,workers%20self%2Didentify%20as%20Hispanic.

